# The genome sequence of the Grey Sedge caddis fly, *Odontocerum albicorne *(Scopoli, 1769)

**DOI:** 10.12688/wellcomeopenres.20124.1

**Published:** 2023-10-12

**Authors:** Sue Skipp, Ian Wallace, Natural History Museum Genome Acquisition Lab, Darwin Tree of Life Barcoding collective, Wellcome Sanger Institute Tree of Life programme, Wellcome Sanger Institute Scientific Operations: DNA Pipelines collective, Tree of Life Core Informatics collective, Darwin Tree of Life Consortium

**Affiliations:** 1 Environment Agency, Rochester, England, UK; 2 British Caddis Recording Scheme, Wirral, England, UK

**Keywords:** Odontocerum albicorne, Grey Sedge caddis fly, genome sequence, chromosomal, Trichoptera

## Abstract

We present a genome assembly from an individual male *Odontocerum albicorne* (the Grey Sedge caddis fly; Arthropoda; Insecta; Trichoptera; Odontoceridae). The genome sequence is 1,287.3 megabases in span. Most of the assembly is scaffolded into 31 chromosomal pseudomolecules, including the Z sex chromosome. The mitochondrial genome has also been assembled and is 16.57 kilobases in length.

## Species taxonomy

Eukaryota; Metazoa; Eumetazoa; Bilateria; Protostomia; Ecdysozoa; Panarthropoda; Arthropoda; Mandibulata; Pancrustacea; Hexapoda; Insecta; Dicondylia; Pterygota; Neoptera; Endopterygota; Amphiesmenoptera; Trichoptera; Integripalpia; Brevitentoria; Leptoceroidea; Odontoceridae; Odontocerinae; *Odontocerum*; *Odontocerum albicorne* (Scopoli, 1769) (NCBI:txid446452).

## Background

**Figure 1.**
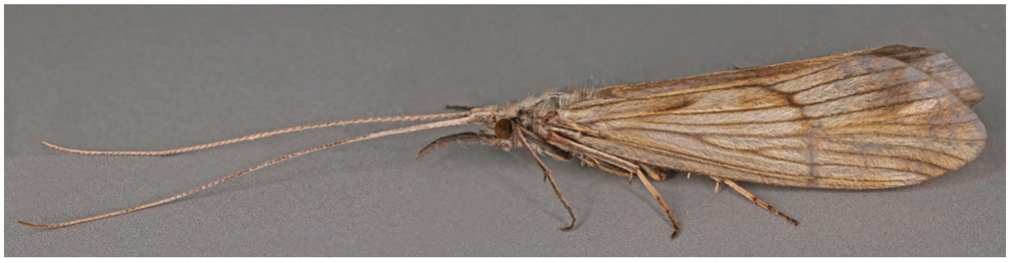
Photograph of adult *Odontocerum albicorne* (not the specimen used for genome sequencing) by Janet Graham, CC-BY.

The adult of *Odontocerum albicorne* ( Figure 1) has the common name of Silver or Grey Sedge, although the male has yellowish wings. Those are held partially rolled around the body when at rest. Adults also have toothed antennae, but the purpose of that is not known. Larvae live in permanent, moderate to fast flowing, streams and rivers on a substratum of stones sand and gravel. The species is absent from Shetland and the Outer Hebrides and scarce in East Anglia due to lack of suitable flowing waters. It is the only member of its family in Britain and Ireland.

The distinctive adults can be easily identified in the wild, with the assistance of Wallace *et al.* (2022). The curved sand grain cases with their unique posterior closure are also easy to recognise and the pupal cases persist for some time after the adult has emerged. Larger larvae have a characteristic anchor mark on their pale heads. Larvae of all sizes can be identified using Wallace *et al.*, 2003).

There is consensus that in Britain the life cycle takes one year but due to a long flight period, from May to September, the larvae are at various sizes throughout. Eggs are laid by females dropping an egg mass into slowly flowing water. The egg absorbs water and expands so the final jelly egg mass, which has a sculptured outer surface, is between 8 and 10 mm in diameter. The eggs do not have any delayed hatching.

The larva spends the day under stones and forages, especially in the early part of the night. Out of preference it is predaceous and eats animals it can subdue or find dead; it has been reported feeding on the eggs of the Bullhead *Cottus gobio* Linnaeus. However, it will also eat plant material if higher energy food is not available. The larva has several interesting features relating to its method of case construction. Other cased caddis join case particles with silk threads and then line the resulting tube with a layer of smooth silk. *O. albicorne* larvae firmly cement the outer case particles together with silk material dispensing with an internal silk lining. The case is very strong and can resist attack from some biting fish. However, if bent, it will snap rather than deform. Other caddis larvae have sharply pointed anal proleg claws that can grip the internal silk lining of the case and help prevent the larva being forcibly removed, but the claws of *O. albicorne* are blunt, with the result that they grip the knobbly inner surface of the case better. The posterior of the case is closed, with a large sand grain attached to the rim of the case, with holes in the attachment to allow for water movement.

This is one of a handful of caddis larvae that have an ichneumonid parasite. *Agriotypus armatus* Curtis normally attacks goerid caddis pupae, but has this species as an alternative host ( Grenier, 1970).

The genome of *O. albicorne* was sequenced as part of the Darwin Tree of Life Project, a collaborative effort to sequence all named eukaryotic species in the Atlantic Archipelago of Britain and Ireland.

## Genome sequence report

The genome was sequenced from one *Odontocerum albicorne* ( Figure 1) collected from River Itchen, Itchen Stoke, UK (51.09, –1.20). A total of 25-fold coverage in Pacific Biosciences single-molecule HiFi long reads was generated. Primary assembly contigs were scaffolded with chromosome conformation Hi-C data. Manual assembly curation corrected 329 missing joins or mis-joins and removed 24 haplotypic duplications, reducing the assembly length by 0.15% and the scaffold number by 7.04%, and increasing the scaffold N50 by 1.45%.

**Figure 2.**
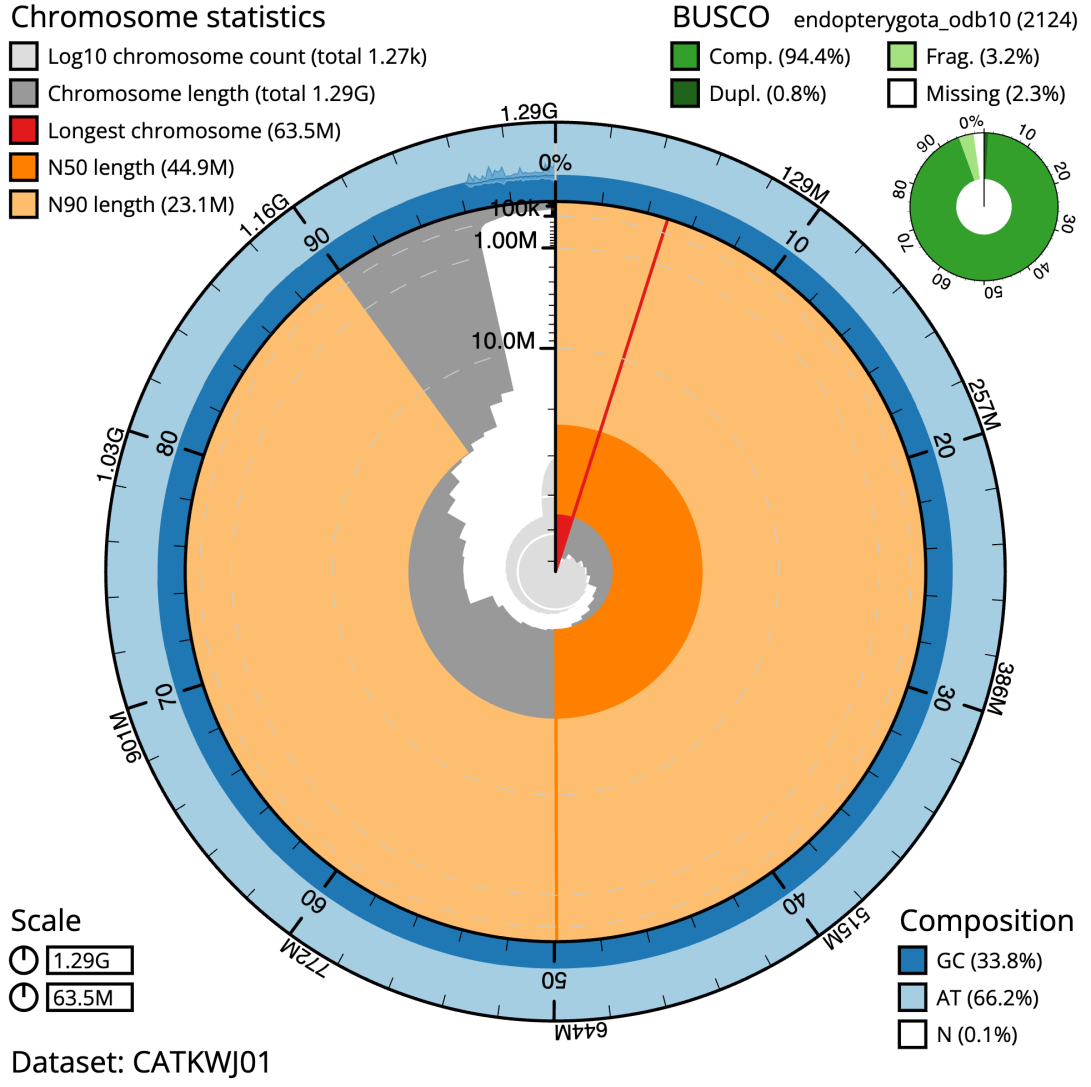
Genome assembly of *Odontocerum albicorne*, iiOdoAlbi1.1: metrics. The BlobToolKit Snailplot shows N50 metrics and BUSCO gene completeness. The main plot is divided into 1,000 size-ordered bins around the circumference with each bin representing 0.1% of the 1,287,343,752 bp assembly. The distribution of scaffold lengths is shown in dark grey with the plot radius scaled to the longest scaffold present in the assembly (63,503,693 bp, shown in red). Orange and pale-orange arcs show the N50 and N90 scaffold lengths (44,898,628 and 23,064,039 bp), respectively. The pale grey spiral shows the cumulative scaffold count on a log scale with white scale lines showing successive orders of magnitude. The blue and pale-blue area around the outside of the plot shows the distribution of GC, AT and N percentages in the same bins as the inner plot. A summary of complete, fragmented, duplicated and missing BUSCO genes in the endopterygota_odb10 set is shown in the top right An interactive version of this figure is available at https://blobtoolkit.genomehubs.org/view/Odontocerum%20albicorne/dataset/CATKWJ01/snail.

**Figure 3.**
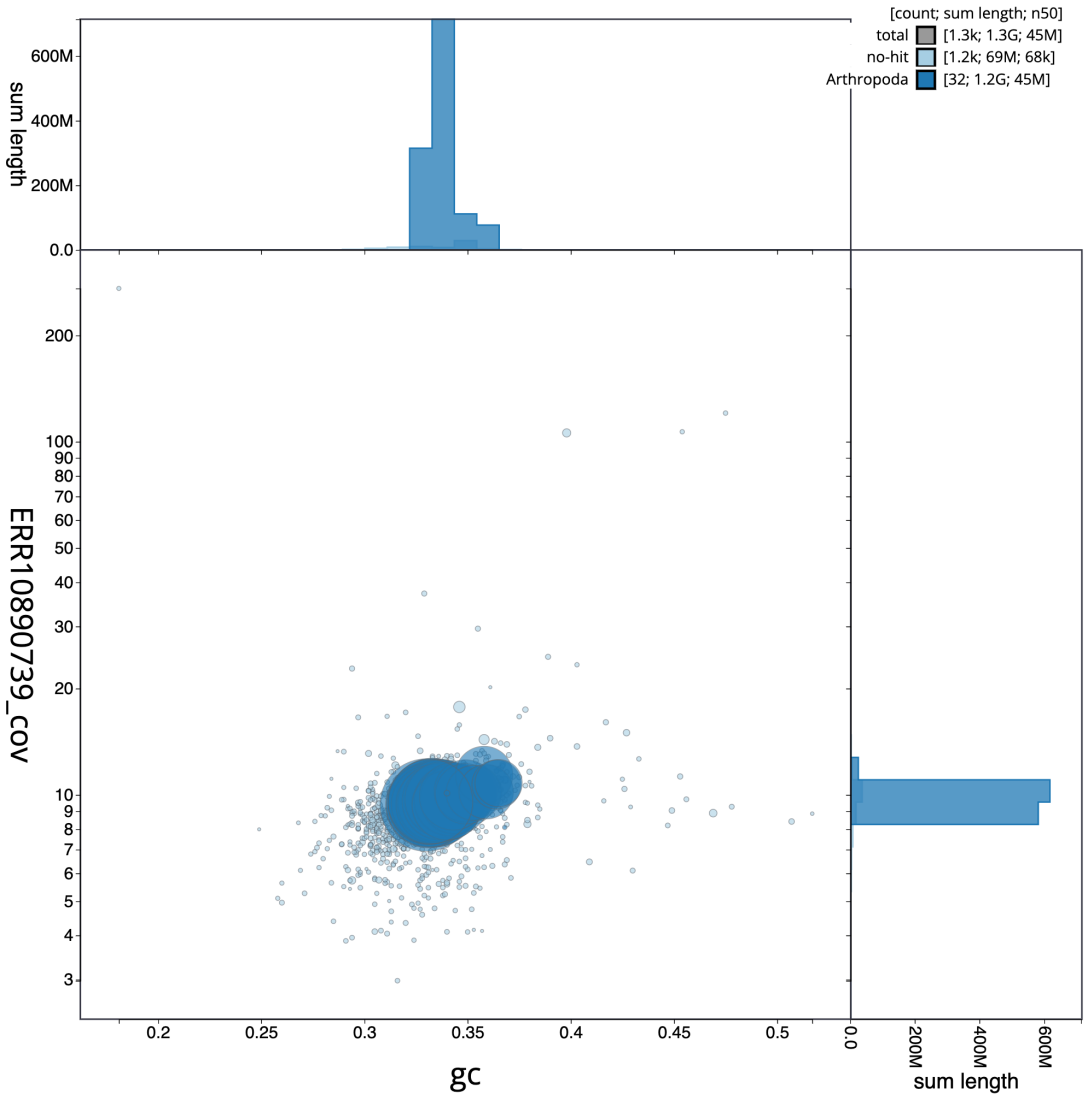
Genome assembly of *Odontocerum albicorne*, iiOdoAlbi1.1: BlobToolKit GC-coverage plot. Scaffolds are coloured by phylum. Circles are sized in proportion to scaffold length. Histograms show the distribution of scaffold length sum along each axis. An interactive version of this figure is available at https://blobtoolkit.genomehubs.org/view/Odontocerum%20albicorne/dataset/CATKWJ01/blob.

**Figure 4.**
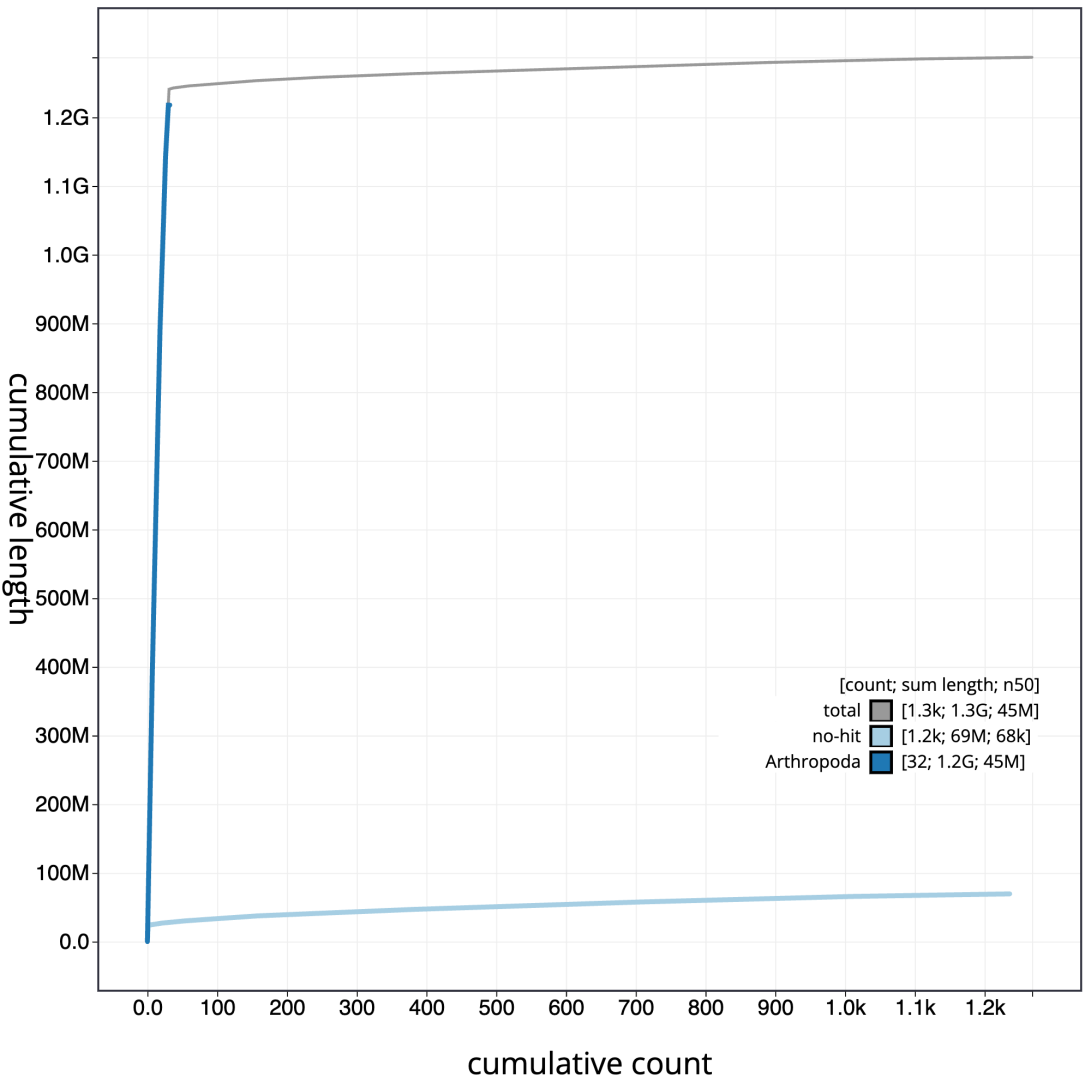
Genome assembly of *Odontocerum albicorne*, iiOdoAlbi1.1: BlobToolKit cumulative sequence plot. The grey line shows cumulative length for all scaffolds. Coloured lines show cumulative lengths of scaffolds assigned to each phylum using the buscogenes taxrule. An interactive version of this figure is available at https://blobtoolkit.genomehubs.org/view/Odontocerum%20albicorne/dataset/CATKWJ01/cumulative.

**Figure 5.**
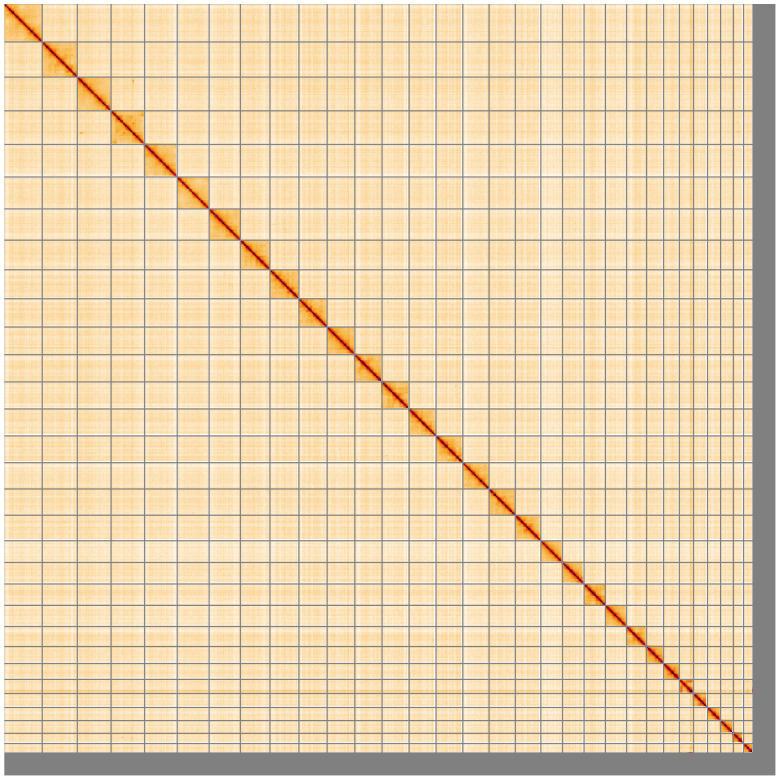
Genome assembly of *Odontocerum albicorne*, iiOdoAlbi1.1: Hi-C contact map of the iiOdoAlbi1.1 assembly, visualised using HiGlass. Chromosomes are shown in order of size from left to right and top to bottom. An interactive version of this figure may be viewed at https://genome-note-higlass.tol.sanger.ac.uk/l/?d=QqP4gJm9SsCzzU461RBsHA.

The final assembly has a total length of 1,287.3 Mb in 1267 sequence scaffolds with a scaffold N50 of 44.9 Mb ( Table 1). A summary of the assembly statistics is shown in Figure 2, while the distribution of assembly scaffolds on GC proportion and coverage is shown in Figure 3. The cumulative assembly plot in Figure 4 shows curves for subsets of scaffolds assigned to different phyla. Most (96.62%) of the assembly sequence was assigned to 10 chromosomal-level scaffolds, representing 30 autosomes and the Z sex chromosome. Chromosome Z was assigned by synteny to *Glyphotaelius pellucidus* (GCA_936435175.1) ( McSwan *et al.*, 2023). Chromosome-scale scaffolds confirmed by the Hi-C data are named in order of size ( Figure 5; Table 2). While not fully phased, the assembly deposited is of one haplotype. Contigs corresponding to the second haplotype have also been deposited. The mitochondrial genome was also assembled and can be found as a contig within the multifasta file of the genome submission.

**Table 1.** Genome data for *Odontocerum albicorne*, iiOdoAlbi1.1.

Project accession data		
Assembly identifier	iiOdoAlbi1.1	
Species	*Odontocerum albicorne*	
Specimen	iiOdoAlbi1	
NCBI taxonomy ID	446452	
BioProject	PRJEB59794	
BioSample ID	SAMEA7520808	
Isolate information	iiOdoAlbi1, male: terminal body (DNA and Hi-C sequencing)	
Assembly metrics *		*Benchmark*
Consensus quality (QV)	57.7	*≥ 50*
*k*-mer completeness	99.99%	*≥ 95%*
BUSCO **	C:94.4%[S:93.6%,D:0.8%], F:3.2%,M:2.3%,n:2,124	*C ≥ 95%*
Percentage of assembly mapped to chromosomes	96.62%	*≥ 95%*
Sex chromosomes	Z chromosome	*localised homologous pairs*
Organelles	Mitochondrial genome assembled	*complete single alleles*
Raw data accessions		
PacificBiosciences SEQUEL II	ERR10880461, ERR10879934	
Hi-C Illumina	ERR10890741	
Genome assembly		
Assembly accession	GCA_949825065.1	
*Accession of alternate haplotype*	GCA_949825055.1	
Span (Mb)	1,287.3	
Number of contigs	6038	
Contig N50 length (Mb)	0.4	
Number of scaffolds	1267	
Scaffold N50 length (Mb)	44.9	
Longest scaffold (Mb)	63.5	

* Assembly metric benchmarks are adapted from column VGP-2020 of “Table 1: Proposed standards and metrics for defining genome assembly quality” from ( Rhie *et al.*, 2021). ** BUSCO scores based on the endopterygota_odb10 BUSCO set using v5.3.2. C = complete [S = single copy, D = duplicated], F = fragmented, M = missing, n = number of orthologues in comparison. A full set of BUSCO scores is available at https://blobtoolkit.genomehubs.org/view/Odontocerum%20albicorne/dataset/CATKWJ01/busco.

**Table 2.** Chromosomal pseudomolecules in the genome assembly of *Odontocerum albicorne*, iiOdoAlbi1.

INSDC accession	Chromosome	Length (Mb)	GC%
OX463838.1	1	63.5	33.0
OX463839.1	2	58.15	33.5
OX463841.1	4	55.89	33.5
OX463840.1	3	55.65	33.5
OX463842.1	5	53.85	33.0
OX463844.1	6	51.78	33.0
OX463845.1	7	48.92	33.5
OX463846.1	8	48.05	33.0
OX463847.1	9	46.77	33.5
OX463848.1	10	45.87	34.0
OX463849.1	11	45.0	33.5
OX463850.1	12	44.9	33.0
OX463851.1	13	44.53	33.5
OX463852.1	14	44.34	33.5
OX463854.1	16	43.56	33.5
OX463853.1	15	43.56	33.5
OX463855.1	17	42.23	34.0
OX463856.1	18	35.88	34.0
OX463858.1	20	35.61	34.5
OX463857.1	19	35.53	34.0
OX463859.1	21	34.99	34.0
OX463860.1	22	33.13	34.5
OX463861.1	23	28.09	35.0
OX463862.1	24	26.23	35.0
OX463863.1	25	23.51	36.0
OX463864.1	26	23.06	35.0
OX463865.1	27	21.7	35.5
OX463866.1	28	21.04	36.0
OX463867.1	29	16.94	36.5
OX463868.1	30	15.61	36.5
OX463843.1	Z	52.95	33.0
OX463869.1	MT	0.02	18.0

The estimated Quality Value (QV) of the final assembly is 57.7 with *k*-mer completeness of 99.99%, and the assembly has a BUSCO v5.3.2 completeness of 94.4% (single = 93.6%, duplicated = 0.8%), using the endopterygota_odb10 reference set ( *n* = 2,124).

Metadata for specimens, spectral estimates, sequencing runs, contaminants and pre-curation assembly statistics can be found at https://links.tol.sanger.ac.uk/species/446452.

## Methods

### Sample acquisition and nucleic acid extraction

A male *Odontocerum albicorne* (specimen ID NHMUK014361308, individual iiOdoAlbi1) was collected from River Itchen, Itchen Stoke, UK (latitude 51.09, longitude –1.20) on 2019-03-19 using a kicknet. The specimen was collected by Sue Skipp (Environment Agency) and identified by Ian Wallace, and then snap-frozen using a dry shipper.

The iiOdoAlbi1 sample was prepared for DNA extraction at the Tree of Life laboratory, Wellcome Sanger Institute (WSI). The specimen was weighed and dissected on dry ice with tissue set aside for Hi-C sequencing. Head and thorax tissue was disrupted using a Nippi Powermasher fitted with a BioMasher pestle. DNA was extracted at the WSI Scientific Operations core using the Qiagen MagAttract HMW DNA kit, according to the manufacturer’s instructions.

### Sequencing

Pacific Biosciences HiFi circular consensus DNA sequencing libraries were constructed according to the manufacturers’ instructions. DNA sequencing was performed by the Scientific Operations core at the WSI on a Pacific Biosciences SEQUEL II (HiFi) instrument. Hi-C data were also generated from terminal body tissue of iiOdoAlbi1 using the Arima2 kit and sequenced on the HiSeq X Ten instrument.

### Genome assembly, curation and evaluation

Assembly was carried out with Hifiasm ( Cheng *et al.*, 2021) and haplotypic duplication was identified and removed with purge_dups ( Guan *et al.*, 2020). The assembly was then scaffolded with Hi-C data ( Rao *et al.*, 2014) using YaHS ( Zhou *et al.*, 2023). The assembly was checked for contamination and corrected using the gEVAL system ( Chow *et al.*, 2016) as described previously ( Howe *et al.*, 2021). Manual curation was performed using gEVAL, HiGlass ( Kerpedjiev *et al.*, 2018) and Pretext ( Harry, 2022). The mitochondrial genome was assembled using MitoHiFi ( Uliano-Silva *et al.*, 2023), which runs MitoFinder ( Allio *et al.*, 2020) or MITOS ( Bernt *et al.*, 2013) and uses these annotations to select the final mitochondrial contig and to ensure the general quality of the sequence.

A Hi-C map for the final assembly was produced using bwa-mem2 ( Vasimuddin *et al.*, 2019) in the Cooler file format ( Abdennur & Mirny, 2020). To assess the assembly metrics, the *k*-mer completeness and QV consensus quality values were calculated in Merqury ( Rhie *et al.*, 2020). This work was done using Nextflow ( Di Tommaso *et al.*, 2017) DSL2 pipelines “sanger-tol/readmapping” ( Surana *et al.*, 2023a) and “sanger-tol/genomenote” ( Surana *et al.*, 2023b). The genome was analysed within the BlobToolKit environment ( Challis *et al.*, 2020) and BUSCO scores ( Manni *et al.*, 2021; Simão *et al.*, 2015) were calculated.

Table 3 contains a list of relevant software tool versions and sources.

**Table 3.** Software tools: versions and sources.

Software tool	Version	Source
BlobToolKit	4.1.7	https://github.com/blobtoolkit/blobtoolkit
BUSCO	5.3.2	https://gitlab.com/ezlab/busco
gEVAL	N/A	https://geval.org.uk/
Hifiasm	0.16.1-r375	https://github.com/chhylp123/hifiasm
HiGlass	1.11.6	https://github.com/higlass/higlass
Merqury	MerquryFK	https://github.com/thegenemyers/MERQURY.FK
MitoHiFi	2	https://github.com/marcelauliano/MitoHiFi
PretextView	0.2	https://github.com/wtsi-hpag/PretextView
purge_dups	1.2.3	https://github.com/dfguan/purge_dups
sanger-tol/genomenote	v1.0	https://github.com/sanger-tol/genomenote
sanger-tol/readmapping	1.1.0	https://github.com/sanger-tol/readmapping/tree/1.1.0
YaHS	1.2a	https://github.com/c-zhou/yahs

### Wellcome Sanger Institute – Legal and Governance

The materials that have contributed to this genome note have been supplied by a Darwin Tree of Life Partner. The submission of materials by a Darwin Tree of Life Partner is subject to the **‘Darwin Tree of Life Project Sampling Code of Practice’**, which can be found in full on the Darwin Tree of Life website here. By agreeing with and signing up to the Sampling Code of Practice, the Darwin Tree of Life Partner agrees they will meet the legal and ethical requirements and standards set out within this document in respect of all samples acquired for, and supplied to, the Darwin Tree of Life Project. 

Further, the Wellcome Sanger Institute employs a process whereby due diligence is carried out proportionate to the nature of the materials themselves, and the circumstances under which they have been/are to be collected and provided for use. The purpose of this is to address and mitigate any potential legal and/or ethical implications of receipt and use of the materials as part of the research project, and to ensure that in doing so we align with best practice wherever possible. The overarching areas of consideration are:

• Ethical review of provenance and sourcing of the material

• Legality of collection, transfer and use (national and international) 

Each transfer of samples is further undertaken according to a Research Collaboration Agreement or Material Transfer Agreement entered into by the Darwin Tree of Life Partner, Genome Research Limited (operating as the Wellcome Sanger Institute), and in some circumstances other Darwin Tree of Life collaborators.

## Data Availability

European Nucleotide Archive: *Odontocerum albicorne* (grey sedge). Accession number PRJEB59794; https://identifiers.org/ena.embl/PRJEB59794. ( Wellcome Sanger Institute, 2023) The genome sequence is released openly for reuse. The *Odontocerum albicorne* genome sequencing initiative is part of the Darwin Tree of Life (DToL) project. All raw sequence data and the assembly have been deposited in INSDC databases. The genome will be annotated using available RNA-Seq data and presented through the Ensembl pipeline at the European Bioinformatics Institute. Raw data and assembly accession identifiers are reported in Table 1.

## Author information

Members of the Natural History Museum Genome Acquisition Lab are listed here: https://doi.org/10.5281/zenodo.4790042.

Members of the Darwin Tree of Life Barcoding collective are listed here: https://doi.org/10.5281/zenodo.4893703.

Members of the Wellcome Sanger Institute Tree of Life programme are listed here: https://doi.org/10.5281/zenodo.4783585.

Members of Wellcome Sanger Institute Scientific Operations: DNA Pipelines collective are listed here: https://doi.org/10.5281/zenodo.4790455.

Members of the Tree of Life Core Informatics collective are listed here: https://doi.org/10.5281/zenodo.5013541.

Members of the Darwin Tree of Life Consortium are listed here: https://doi.org/10.5281/zenodo.4783558.
