# Supplementary material for: The genome sequence of the Grey Sedge caddis fly, Odontocerum albicorne (Scopoli, 1769)
Source: Wellcome Open Res. 2024 May 22;8:445. Originally published 2023 Oct 12. [Version 2] doi: 10.12688/wellcomeopenres.20124.2 (PMC11112305; doi:10.12688/wellcomeopenres.20124.2)
Supplement: Peer review history

# Reviewer report: Reviewer response for version 1

**DOI:** 10.21956/wellcomeopenres.22286.r78134

**Published:** 2024-05-22

**Authors:** Kevin M Moran

**Affiliations:** 1 Biology, The University of Memphis (Ringgold ID: 5415), Memphis, Tennessee, USA

I am glad to see your genome assembly of *Odontocerum albicorne*. Overall, I found your manuscript to be well written and your methods sound. I was able to follow your assembly and annotation processes. I believe this genome will serve as a valuable reference for future research on Trichoptera.

 One thing to note is that I suspect your BUSCO completeness is under the true value. There is a single low quality Trichopteran representative in the endopterygota_odb10 reference set. A lack of a close relative will cause the pipeline to overlook valid matches.

Are sufficient details of methods and materials provided to allow replication by others?

Yes

Is the rationale for creating the dataset(s) clearly described?

Yes

Are the datasets clearly presented in a useable and accessible format?

Yes

Are the protocols appropriate and is the work technically sound?

Yes

Reviewer Expertise:

Phylogenetics, Bioinformatics, Genomic assembly.

I confirm that I have read this submission and believe that I have an appropriate level of expertise to confirm that it is of an acceptable scientific standard.

# Reviewer report: Reviewer response for version 1

**DOI:** 10.21956/wellcomeopenres.22286.r80906

**Published:** 2024-05-10

**Authors:** John Soghigian, Huiqing Yeo

**Affiliations:** 1 University of Calgary, Calgary, Alberta, Canada; 2 Faculty of Veterinary Medicine, University of Calgary, Calgary, AB, Canada

Skipp *et al.* present an excellent genome for the grey sedge caddis fly. The authors provide a detailed background on the species, and summarize the genome they generated well. Overall, this is a superb genome note, but we have a couple of minor comments:

- In the background, the authors state, "There is consensus that in Britain the life cycle takes one year but due to a long flight period, from May to September, the larvae are at various sizes throughout." It would be good to define what is long flight period here.
- Again in the background, the authors state, "Out of preference it is predaceous and eats animals it can subdue or find dead". The phrasing of this sentence is slightly confusing - does this mean that the larvae are predacious but are also scavengers? 
- Regarding larval behavior, the authors say: "The larva spends the day under stones and forages,...", are there any relevant citations for the life history traits described here?
- In describing the genome, there is one confusing sentence: "Most (96.62%) of the assembly sequence was assigned to 10 chromosomal-level scaffolds, representing 30 autosomes and the Z sex chromosome." Could the authors provide more information on this? Was ten meant to be 31?

Overall this is an excellent genome report, despite our minor comments, above.

Are sufficient details of methods and materials provided to allow replication by others?

Yes

Is the rationale for creating the dataset(s) clearly described?

Yes

Are the datasets clearly presented in a useable and accessible format?

Yes

Are the protocols appropriate and is the work technically sound?

Yes

Reviewer Expertise:

Genomics, evolutionary biology

We confirm that we have read this submission and believe that we have an appropriate level of expertise to confirm that it is of an acceptable scientific standard.
